# Editorial: Novel therapeutic strategies for the treatment of respiratory diseases

**DOI:** 10.3389/fphar.2023.1340116

**Published:** 2023-12-06

**Authors:** Serena Di Vincenzo, Maria Ferraro, Maria Letizia Manca, Josè Esteban Peris, Elisabetta Pace

**Affiliations:** ^1^ Institute of Translational Pharmacology (IFT), National Research Council of Italy (CNR), Palermo, Italy; ^2^ Department of Life and Environmental Sciences of the University of Cagliari, Cagliari, Italy; ^3^ Faculty of Pharmacy and Pharmaceutical Technology and Parasitology, University of Valencia, Valencia, Spain

**Keywords:** respiratory diseases, chronic inflammation (CI), drug delivery, innovative treatments, nanotechnologies

Chronic respiratory diseases including asthma, Chronic Obstructive Pulmonary Disease (COPD) and cystic fibrosis are listed among the leading causes of disability and death worldwide. The management of these chronic respiratory diseases represents a serious problem for the public health system (Heron, 2019; Khaltaev and Axelrod, 2019).

The purpose of this Research Topic was to collect original basic and translational research (*n* = 5) and review articles (*n* = 2) focused on the use of innovative therapeutic strategies for the treatment of respiratory diseases but also on the study of the molecular signaling pathways involved in the pathogenesis of these diseases and the identification of new target therapy.

Respiratory diseases are defined by lung function impairment typically caused by chronic or acute injury. Cigarette smoke, allergens, environmental pollutants but also genetic mutations are all determinants of these diseases (Khaltaev and Axelrod, 2019; Han et al., 2023). The prevention and non-exposure to the main risk factors is the only way to avoid the development of these diseases. The treatment of some chronic respiratory diseases, such as asthma and COPD, only allows to cure the symptoms and delay the progression of the disease towards more serious forms (Christenson et al., 2022; Reddel et al., 2022; Han et al., 2023). Patients affected by these pathologies do not recover and are forced to use anti-inflammatory and bronchodilators drugs for life with the consequent development of cortico-resistance and serious side effects (Wadhwa et al., 2019; Rahmawati et al., 2021). To date the treatments for chronic respiratory diseases are particularly challenging because the traditional drugs are often not effective. One original research article published in this Research Topic, was focused on COPD. In detail, Milad et al. have investigated the impact of smoking, the main risk factor of COPD, on the efficacy of new immunomodulatory therapies for COPD patients. The study demonstrated, using a mouse model, that the use of immunomodulators, such as anti-IL1, during exposure to cigarette smoke could interfere with lung adaptation while the administration of the drug during smoking cessation would accelerate the resolution of inflammation. This highlights that in clinical trials of new biologics for the treatment of COPD, it is important to take into account the patient’s smoking status and treatment timing.

As above mentioned, also the exposure to environmental pollutants can promote lung injury. Tang et al. found, through studies of metabolomics, potential biomarkers correlated with oxidative stress and inflammation caused by air pollution (PM_2.5_). The Authors also administrated Yangyinqingfei Decoction (YYQFD), a drug used to treat some respiratory disease together with the traditional drug, to PM_2.5_ exposed mice. Their results showed that this drug could counteract the effects of PM_2.5_ exposure suppressing inflammatory factors and oxidative stress levels and reshaping unsaturated fatty acid metabolism. Tanner et al. instead, focused their attention on asthma starting from previous observation regarding DNA lesions caused by the increased release of reactive oxygen species (ROS) by the eosinophils. These chromatin lesions are mainly repaired by DNA 8-oxoG glycosylase (OGG1), the silencing of which has been shown to reduce allergic inflammatory responses. For this, they investigated the effect of TH5487, a small molecule that interferes with the binding of OGG1 to DNA, in a mouse model of allergen-induced airway inflammation. Administration of TH5487 decreased the recruitment of immune cells to the lungs and mucus accumulation in the small airways. These results suggest that OGG1 could be a novel therapeutic target of allergic asthma.

A comprehensive view of cystic fibrosis gene therapy is provided with the review of Sui et al. Cystic fibrosis is caused by mutations in a single gene encoding cystic fibrosis transmembrane conductance regulator (CFTR). It was one of the first diseases to be considered for gene therapy and despite this, clinical trials have not yet given the desired results (Grasemann and Ratjen, 2023). This review, retrace the history of gene therapy for cystic fibrosis, underlining the difficulties encountered in the first clinical trials and how these have been overcome in the last decade thanks to the use of viral vectors for transduction in airways and more accurate animal models, up to the most advanced gene editing strategies such as CRISPR/Cas-nuclease approach.

Respiratory failure can also be the result of an acute lung injury (ALI) (Dutta et al., 2023). Two original research articles here studied the two main pathophysiological aspects of the Acute lung injury (ALI): injury to the vascular endothelium and to alveolar epithelium. The study of Min et al. investigated whether Tetramethylpyrazine (TMP) could have beneficial effects on pulmonary vascular endothelial barrier dysfunction, a key pathologic process of ALI. Treatment of a mouse model of ALI with this drug showed a decrease of inflammatory cell infiltration and damage in lung tissue, an inhibition of Rac1/LIMK1/ZO-1/occludin signaling pathway. Therefore, TMP protects the pulmonary microvascular endothelial cell barrier and may have a promising therapeutic role in preventing acute lung injury from sepsis. Instead, Kong et al. studied another pathophysiological change in ALI, the damage of alveolar epithelial cells (AECs) caused by uncontrolled inflammation. In their study, the administration of Fibroblast growth factor 10 (FGF10) to mice with inducted ALI seems to be a valid strategy for the treatment of ALI. FGF10 activates autophagy through the increase of BMP4 expression and induces alveolar epithelial cell regeneration preserving the integrity of pulmonary epithelial cell function.

Given that, it becomes increasingly important to study the pathogenetic mechanisms of respiratory diseases to identify new therapeutic targets or to find new strategies for the specific delivery of traditional or new drugs. This would lead to: low doses of drug, increased bioavailability and controlled release of the incorporated drug with a reduction of drug resistance and side effects. In this regard, Taghavizadeh Yazdi et al. reviewed the literature with the aim of summarizing the different types and the main materials used for nanoparticle-based drugs, the main system of drug delivery and the *in vitro*, *in vivo* and clinical trial studies on their efficacy in the treatment of different respiratory diseases. The review showed that nanoparticle-based medicine is a promising tool, but further studies are still needed.

In conclusion, this Research Topic has highlighted how knowledge on respiratory diseases is constantly growing and how animal models can help the understanding of the pathophysiological aspects of the main respiratory diseases as well as the pre-clinical testing of new therapeutic approaches. Future studies are needed to translate these results to human diseases to improve patients’ living conditions.
